# Regulation of immune checkpoints by electronic cigarette

**DOI:** 10.3389/fonc.2026.1772432

**Published:** 2026-03-12

**Authors:** Ana Gabriela Leija-Montoya, Sandra Castillo-Uribe, Javier González-Ramírez, Mario Alberto Isiordia-Espinoza, Idanya Serafin-Higuera, Gustavo Martínez-Coronilla, José Gustavo Vázquez-Jiménez, Nicolás Serafín-Higuera

**Affiliations:** 1 Facultad de Medicina y Nutrición, Universidad Autónoma de Baja California, Mexicali, Mexico; 2 Laboratorio de Biología Celular, Centro de Ciencias de la Salud Mexicali, Facultad de Odontología Mexicali, Universidad Autónoma de Baja California, Mexicali, Mexico; 3 Laboratorio de Biología Molecular, Centro de Ciencias de la Salud Mexicali, Facultad de Enfermería Mexicali, Universidad Autónoma de Baja California, Mexicali, Mexico; 4 Instituto de Investigación en Ciencias Médicas, Departamento de Clínicas, División de Ciencias Biomédicas, Centro Universitario de los Altos, Universidad de Guadalajara, Tepatitlán de Morelos, Mexico; 5 Facultad de Medicina y Psicología, Universidad Autónoma de Baja California, Tijuana, Mexico

**Keywords:** aerosols, cancer, e-cigarette, e-liquid, immune checkpoints, immunoregulation, nicotine, vaping

## Abstract

An e-cigarette device consists of a cartridge that stores e-liquid, a mouthpiece, a heating element, and a power source. The e-liquid is heated by a coil and a wick, generating an aerosol that is inhaled by the user. Although some sectors promote e-cigarettes as a smoking cessation tool, increasing scientific evidence associates their use with adverse health effects, including cytotoxic, genotoxic, potentially carcinogenic effects, and immunological alterations. Immune checkpoints are a group of molecules with co-stimulatory or co-inhibitory functions that bind their ligands to activate or suppress intracellular signaling pathways in T cells. Inhibitory immune checkpoints are expressed on immune cells and regulate the magnitude of immune activation to prevent excessive immune responses. Several studies have reviewed the effects of cigarette smoke and its components on immune checkpoints; however, the effects of e-liquids and their aerosols on immune checkpoints have not been comprehensively reviewed. In this work, we aimed to analyze and discuss the contribution of e-cigarette aerosols and core e-liquid components to the regulation of inhibitory immune checkpoint expression and function, as well as their biological impact on cellular processes. Available evidence indicates that e-cigarette aerosols and major e-liquid constituents can modulate the expression and function of diverse inhibitory immune checkpoints in models of various cancers, rheumatoid arthritis, and physical stress. Further studies are required to clarify the biological consequences of immune checkpoint alterations induced by e-cigarette aerosols and their contribution to disease development.

## 1 Introduction

Electronic Nicotine Delivery Systems (ENDS) are devices designed to heat a liquid solution, commonly known as e-liquid (1). The composition of e-liquids varies, containing different concentrations of nicotine (including nicotine-free options) (2), flavoring compounds, propylene glycol, vegetable glycerin, and other additives present in small quantities, to produce an inhaled aerosol. These devices are more commonly referred to as “e-cigarettes” (1, 3). Although some sectors promote e-cigarettes as a smoking cessation tool, growing scientific evidence indicates that their use is associated with adverse health effects, including cytotoxic, genotoxic, and potentially carcinogenic outcomes, as well as immunological alterations (4, 5). E-cigarette aerosols may contribute to the development of chronic inflammatory conditions through multiple mechanisms, affecting the function of various immune cells (5).

Immune checkpoints are a group of molecules with co-stimulatory or co-inhibitory functions that bind their respective ligands to activate or suppress intracellular signaling pathways in T cells (6). Inhibitory immune checkpoints are expressed on immune cells and regulate the magnitude of immune activation to prevent immune overactivation (7). Consequently, these molecules have become targets of cancer immunotherapy, with the aim of enhancing immune cell activation (7).

Several studies have reviewed the effects of cigarette smoke and its components on immune checkpoints, including biological implications and therapeutic relevance (8–12). However, the impact of major components of e-cigarette liquids and aerosols on immune checkpoints and their potential biological effects have not been comprehensively reviewed. Inhibitory immune checkpoints play a central role in development of different disorders, including cancer and autoimmune diseases (13, 14). Consequently, these molecules serve as targets of a novel anticancer drug class called immune checkpoint inhibitors (ICIs) (14, 15). The aim of this work was to analyze and discuss the contribution of e-cigarette aerosols and core e-liquid components to the regulation of inhibitory immune checkpoint expression and function, as well as their biological impact on cellular processes involved in health and disease.

A literature search was conducted between September and October of 2025, with no time restrictions. The PUBMED and SCOPUS databases were searched using combinations of the following terms: electronic nicotine delivery systems, ENDS, e-cigarette, electronic-cigarette, vaping, propylene glycol, glycerin, vanillin, ethyl maltol, ethyl butyrate, butyric acid ethyl ester, cinnamaldehyde, cinnamic aldehyde, PD-L1, CTLA-4, PD-1, LAG-3, TIM-3, TIGIT, BTLA, PD-L2, VISTA, SIRPA, LILRB4, SIGLEC7, SIGLEC15, SIGLEC9, SIGLEC10, CD112R, KIR, NKG2A, CD47, CLEVER-1, HLA-G, SLAMF7, SLAMF4, SLAMF3, CD24, and CD155. Alternatives names and synonyms were also included. Inclusion criteria were English-language publications involving e-cigarette aerosols, propylene glycol, glycerin, nicotine, or commonly used e-liquid flavoring agents in e-liquids, electronic nicotine delivery systems, that modulate the expression and/or function of inhibitory immune checkpoints. Emphasis was placed on identifying inhibitory immune checkpoints regulated by core e-liquid components and aerosols, the associated molecular mechanisms, altered biological processes, disease contexts, and experimental approaches.

## 2 E-cigarette

An e-cigarette device consists of a cartridge or tank for liquid storage, a mouthpiece, a heating element, and a power source. The cartridge contains e-liquid, which is heated by a coil and wick to generate an aerosol inhaled by the user (16). During aerosol generation, increasing coil temperatures can lead to the formation of toxic substances, including aldehydes such as formaldehyde and acetaldehyde, free radicals at significant concentrations (17), and heavy metals such as lead, nickel, and chromium (18). Zhao et al. reported a significant potential carcinogenic risk associated with e-cigarette exposure (19). Furthermore, formaldehyde is classified by the International Agency for Research on Cancer (IARC) as a Group 1 carcinogen (20). Coil resistance and atomizer design influence particle size distribution, thereby affecting lung deposition, systemic bioavailability of compounds, and the number of particles emitted (21, 22). *In vitro* studies indicate that ENDS aerosols can induce oxidative stress, DNA damage, and a type 2 inflammatory response characterized by increased IL-4, IL-5, and IL-13 production (23). Additional effects include epigenetic alterations, the generation of mutagenic compounds, dysregulation of oncogenic signaling pathways (24), and activation of biological processes associated with lung carcinogenesis and tumor metastasis (25, 26), including cell migration, invasion, angiogenesis, and modulation of the tumor microenvironment (TME). At the epigenetic level, e-cigarette use has been shown to induce hypermethylation of oral epithelial DNA, modifying the epigenome of oral epithelial cells, an important factor related to carcinogenesis-related pathways (27).

Since their introduction in 2003 by Hon Lik, e-cigarettes have evolved through four generations to meet growing global demand as alternatives to traditional tobacco products. Concurrently, e-cigarette use has risen markedly among adolescents and young adults. In 2024, an estimated 3.87 million middle and high school students in the US used ENDS (28). Their rapid technological evolution and growing popularity have intensified debate regarding safety, impact on public health, and regulation. The broad range of identified chemical constituents, many of which are associated with adverse effects in humans, animals, and cellular models, highlights the urgent need for stricter regulation and systematic toxicological studies to assess both short- and long-term risks in users and in indirectly exposed populations (28).

## 3 Immune checkpoints

Numerous immune checkpoint molecules have been identified (7). Their functions and regulatory molecular mechanisms have been extensively reviewed (7, 29–31). This section provides an overview of immune checkpoints that have been linked to e-cigarette exposure.

### 3.1 TIGIT

T-cell immunoglobulin and immunoreceptor tyrosine-based inhibitory motif domain (TIGIT) plays a central role in autoimmunity and immune regulation, functioning primarily to limit T cell-mediated inflammation rather than to drive acute T cell priming (32). TIGIT is weakly expressed or absent on naïve lymphocytes, but it can be rapidly induced following antigenic stimulation and other inflammatory signals (32). TIGIT is also expressed on multiple immune cell types relevant to cancer, and its functional role appears to be cell type-dependent. Moreover, elevated TIGIT expression has been associated with poor prognosis in non–small cell lung cancer and has been proposed as a predictive biomarker of reduced chemotherapy response in muscle-invasive bladder cancer. Previous data from other malignancies also suggest that TIGIT contributes to the immunosuppressive TME, promoting tumor growth, and may serve as a prognostic and predictive marker, as well as a therapeutic target in cancer immunotherapy (33). In fact, the first commercially available anti-TIGIT monoclonal antibody tiragolumab was approved by the U.S. Food and Drug Administration in 2021 (33).

### 3.2 CTLA-4

Cytotoxic T-cell-associated protein 4 (CTLA-4) is a T-cell-associated receptor that acts as a critical immune checkpoint. It plays a fundamental role in the T-cell-mediated responses by attenuating co-stimulatory signaling, limiting immunosurveillance, and promoting immune homeostasis (34). CTLA-4 is a key modulator of T cell priming and activation, contributing to peripheral tolerance and preventing uncontrolled T cell proliferation. Increased CTLA-4 expression has been associated with systemic immunoinflammatory disorders, including autoimmunity and cancer (34). Moreover, enhanced CTLA-4-mediated inhibitory signaling can promote immune evasion in malignancies and impact the efficacy of cancer immunotherapy (35).

CTLA-4 may act independently or synergistically with other coinhibitory pathways to support tumorigenesis through direct or indirect mechanisms. The most established mechanism involves CTLA-4 expression on cytotoxic T cells, which competitively binds B7 ligands, resulting in inhibitory signaling that reduces T cell proliferation and cytokine release. This process contributes to suppression of antitumor immune responses, particularly during early stages of tumorigenesis (34). Additional CTLA-4-mediated regulatory mechanisms include the induction of T cell regulatory pathways (35), reverse signaling through indoleamine 2,3-dioxygenase (IDO), and Casitas B-cell lymphoma protein (Cb1-b)-mediated signaling. Overall, CTLA-4 and its therapeutic targeting play central roles in cancer pathogenesis, immunotherapy, and autoimmune disease development (34).

### 3.3 BTLA

The B and T lymphocyte attenuator (BTLA) belongs to the immunoglobulin superfamily, which also includes PD-1, CTLA-4, CD28, and ICOS. BTLA acts as a co-inhibitory immune checkpoint receptor, with structural and functional similarities to CTLA-4 and PD-1. It is widely expressed on the surface of T cells, B cells, monocytes, and natural killer (NK) cells. In patients with cancer, BTLA is highly expressed on tumor-specific T cells, and its positive expression in various tumor-infiltrating lymphocytes (TILs) is associated with poor prognosis (36, 37).

The BTLA ligand, herpesvirus entry mediator (HVEM), induces tyrosine phosphorylation and mediates the recruitment of Src homology 2 (SH2) domain-containing phosphatases 1/2. This leads to inhibition of B and T cell proliferation and activation. Similar to CTLA-4 and PD-1, BTLA functions as an inhibitory immune checkpoint receptor and is expressed by activated T and B cells, subsets of dendritic cells, macrophages, and NK cells. Through HVEM-BTLA binding, BTLA contributes to the suppression of the antitumor immune response and has been reported to be overexpressed in patients with non-small cell lung cancer presenting with lymph node metastases (36, 37).

### 3.4 LAG-3

LAG-3 is a highly complex and multifaceted immune receptor that acts as a coinhibitory molecule on regulatory T cells, suppressing other effector T cell responses. Major histocompatibility complex class II (MHC-II) and fibrinogen-like protein 1 (FGL1) are the principal ligands for LAG-3. In addition, LAG-3 can interact with the T-cell receptor (TCR) CD3 complex to inhibit TCR signal transduction, leading to impaired T cell proliferation and cytokine secretion. The functional activity of LAG-3 depends on dynamic protein dimerization, which influences both ligand binding and association with the TCR (38). Furthermore, soluble LAG-3 protein (sLAG3) may compete with surface LAG-3 interactions and has been proposed as a potential biomarker for predicting outcomes of ICI therapy (38, 39).

Clinical evidence indicates that LAG-3 receptor blockers have a favorable efficacy and safety profile and may offer therapeutic benefit to patients who have exhausted currently available options for histologically confirmed, locally advanced, unresectable, or metastatic solid tumors, particularly when combined with drugs targeting the PD-1 receptor. LAG-3 and PD-1 act as non-redundant inhibitory receptors on exhausted T cells and synergistically suppress T-cell activation. Their co-expression amplifies T-cell dysfunction within the TME beyond the effects of either immune checkpoint alone (38, 39).

### 3.5 PD-1, PDL-1, and PDL-2

The programmed cell death protein 1 (PD-1) receptor and its ligands, programmed death-ligand 1 (PD-L1) and PD-L2, are essential components of the immune checkpoint pathway that regulates immune homeostasis. PD-1 is a type I transmembrane receptor of the CD28 family, primarily expressed on activated T cells, B cells, and NK cells. Binding PD-1 to its ligands, PD-L1 or PD-L2, attenuates TCR signaling, thereby limiting T-cell activation and clonal expansion. PD-L1 is expressed by a wide range of cells, including tumor cells, macrophages, and dendritic cells, and its expression is strongly induced by pro-inflammatory cytokines such as interferon-γ (IFNγ) (40).

The PD-1/PD-L1 axis functions as a major regulator of immune tolerance. In addition to promoting T-cell exhaustion, PD-1/PD-L1 signaling broadly constrains T-cell activation, proliferation, and cytotoxicity capacity. Inhibition of this pathway has been shown to reinvigorate T-cell function, restoring cytokine secretion and cytolytic activity of CD8+ T cells, which are essential for effective tumor clearance and pathogen defense. Clinical and experimental evidence indicate that PD-1 inhibition can restore effective immune responses in chronic infections and cancers, highlighting its therapeutic potential. The FDA has approved immune checkpoint blockade (ICB) therapies for non-small cell lung cancer, which can be administrated alone or in combination (40, 41).

### 3.6 TIM-3

T-cell immunoglobulin and mucin domain 3 (TIM-3) is a cell surface receptor encoded by the HAVCR2 gene that was initially identified on activated CD4+Th1 cells, as well as cytotoxic CD8+T cells. *In vivo* studies using TIM-3 blocking antibodies have shown increased T-cell responses and autoimmunity, supporting its function as an immune checkpoint receptor (42).

TIM-3 plays a regulatory role in immune responses across a wide range of pathological conditions, including cancer, autoimmune diseases, transplant rejection, chronic inflammation, and persistent infections such as hepatitis B virus, hepatitis C virus, human immunodeficiency virus (HIV), and parasitic infections. In addition, TIM-3 has been reported to exert protective effects in drug-induced acute kidney injury and to contribute to the maintenance of immune tolerance during pregnancy (43).

In cancer, TIM-3 expression characterizes the most dysfunctional or terminally exhausted subset of CD8+T cells. Evidence indicates that combined blockade of PD-1 and TIM-3 enhances antitumor activity in multiple tumor models, including colorectal carcinoma and breast cancer. In breast cancer models, TIM-3 inhibition has also promoted CD8+T cell responses to paclitaxel through a mechanism involving dendritic cell-mediated uptake of extracellular DNA and activation of the cyclic GMP/AMP synthase-stimulator of interferon genes (cGAS-STING) pathway. Despite these advances, a substantial proportion of patients with melanoma show little response to ICIs, and relapses remain common among those who initially respond (42).

### 3.7 CD47

Cluster of differentiation 47 (CD47) is an immune checkpoint molecule commonly referred to as the “don’t eat me” signal and has attracted substantial attention due to its role in facilitating tumor immune evasion. Although CD47 is ubiquitously expressed on healthy cells, it is frequently overexpressed in both hematologic malignancies and solid tumors, where it interacts with signal regulatory protein alpha (SIRPα) expressed on macrophages and dendritic cells (44). Engagement of the CD-47-SIRPα axis delivers a strong anti-phagocytic signal that suppresses tumor cell clearance, enabling malignant cells to evade innate immune surveillance despite immunologic recognition (44).

Owing to its upstream position in immune regulation, CD47 has emerged as a promising therapeutic target with the potential to link innate and adaptive immune responses, particularly in immunologically “cold” tumors that exhibit limited T cell infiltration and poor responsiveness to PD-1/PDL1-directed therapies (44). Despite the strong biological rationale, clinical translation of CD47-targeted therapies has proven more challenging than initially anticipated. Development has been limited by on-target, off-tumor toxicities, most notably anemia and thrombocytopenia, along with suboptimal pharmacokinetic profiles and modest efficacy as monotherapy (44). These challenges highlight the biological and clinical complexities of CD47 blockade, as well as strategic limitations related to trial design, patient selection, and combination of strategies.

Nevertheless, the CD47-SIRPα axis remains a compelling target in cancer immunotherapy. Recent clinical setbacks reflect the intricacies of immune modulation rather than a fundamental failure of the target itself. Future success will likely depend on advances in antibody engineering to reduce hematologic toxicity, biomarker-driven patient stratification, and rational combination regimens tailored to tumor-specific immune contexts (44).

### 3.8 CD24

CD24 is a glycosylphosphatidylinositol (GPI)-anchored glycoprotein expressed on the cell surface, with cell type-specific glycosylation determined by the local repertoire of glycosyltransferases. In cancer, dysregulation of these enzymes results in aberrant CD24 glycosylation, contributing to malignant characteristics such as altered cell adhesion, immune evasion, and metastatic potential. CD24 is broadly expressed within the immune system, with higher levels observed in progenitor and metabolically active cells and lower expression in terminally differentiated cells. Notably, CD24 is frequently overexpressed across a wide range of human malignancies, where it functions as a multifunctional receptor involved in diverse cellular processes (45, 46).

CD24 interacts with several ligands, including P-selectin and sialic acid-binding immunoglobulin-like lectins (Siglecs), particularly Siglec-10 in humans. Through complexes involving CD24, Siglec-10, and damage-associated molecular patterns, the CD24/Siglec-10 axis suppresses tissue damage-induced immune responses and facilitates tumor immune escape. Beyond its immunomodulatory role, CD24 can also serve as a receptor for certain pathogens. Clinically, CD24 overexpression has been reported in multiple cancer types, including breast, ovarian, and hepatocellular carcinoma, where it correlates with enhanced tumor invasiveness, metastatic potential, increased proliferation, and activation of oncogenic pathways such as Wnt/β-catenin signaling, underscoring its relevance in cancer pathogenesis and as a potential therapeutic target (45, 46).

## 4 E-cigarette and immune checkpoints

Core components of e-liquid include propylene glycol (approximately 50% of the composition), vegetable glycerin (approximately 40%), nicotine (approximately 1-5%), and flavoring agents (approximately 4%). Although the composition varies by brand and user preference, it also affects the components present in the aerosols generated (16, 28). In this section, the contribution of e-cigarette aerosols and e-liquids components on the regulation of immune checkpoints is reviewed.

### 4.1 E-cigarette aerosols

**Figure 1:**
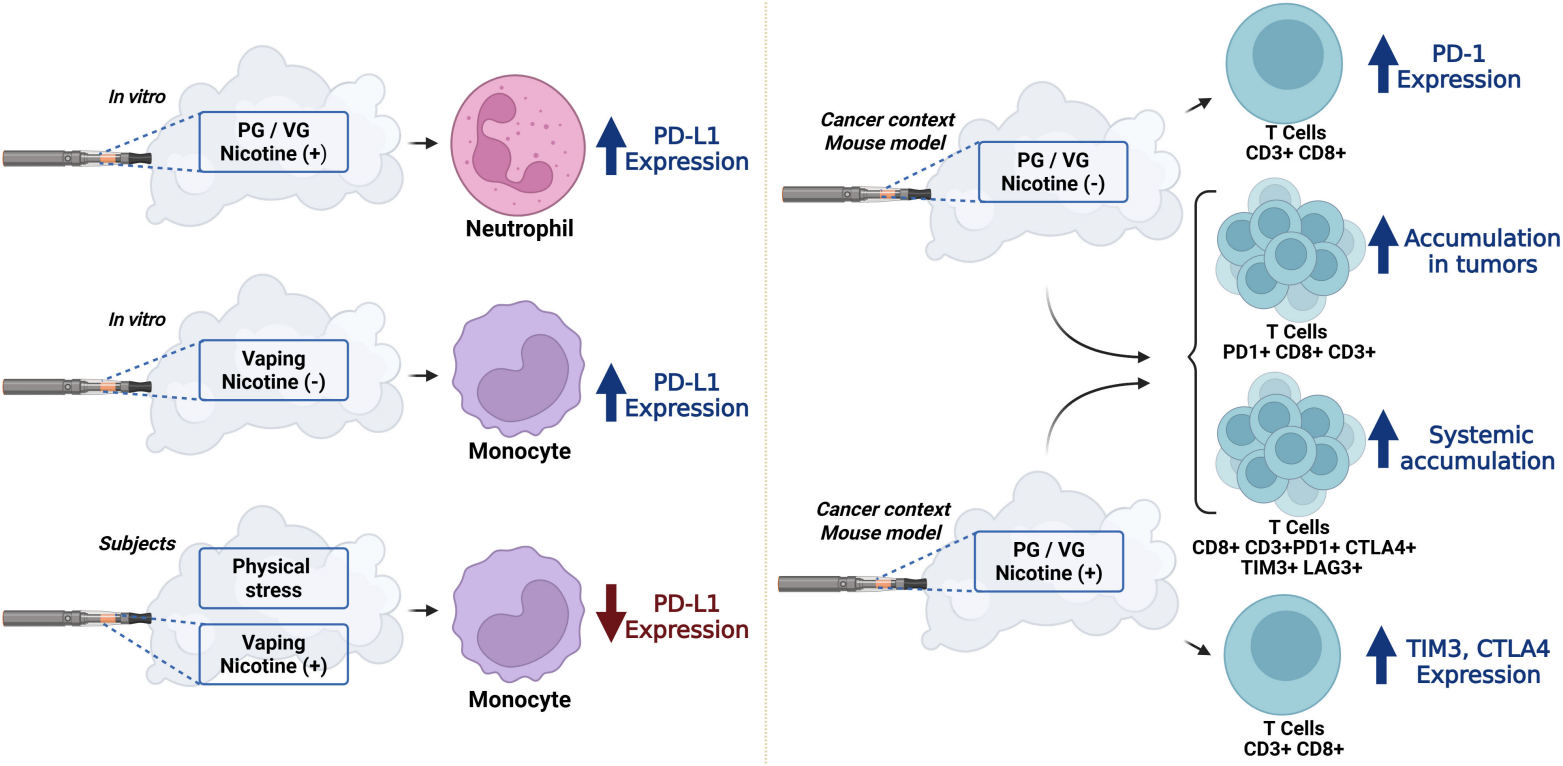
Regulation of immune checkpoints by e-cigarette aerosols. Aerosols from e-liquids with different compositions can alter the expression of diverse immune checkpoints in neutrophils, monocytes, and T cells. In addition, these aerosols can induce local and systemic accumulation of immune checkpoint-expressing T cells in the context of cancer. Propylene glycol (PG); vegetable glycerin (VG). Created in BioRender. Leija, G. (2026) https://BioRender.com/txg4mnn.

Reports indicate that exposure to e-cigarette aerosols can induce changes in the expression of immune checkpoints in diverse cell types across multiple models, including self-reported e-cigarette users, mouse models exposed to e-cigarette aerosols, and *in vitro* models. Moreover, variations in e-liquid composition have been analyzed showing different effects on immune checkpoint expression (**Figure 1**).

Decreased RNA expression levels of immune checkpoints, such as TIGIT, CTLA-4, and BTLA were observed in epithelium obtained from nasal scrape biopsies of self-described active e-cigarette users compared with samples from non-smokers (47). Additionally, a study indicated that e-cigarette aerosols can alter nasal live-attenuated influenza virus–induced immune responses. Gene expression profiles in nasal scrape biopsy samples from self-reported e-cigarette users inoculated with the attenuated virus differed from those in non-smokers, including reduced expression of immune checkpoint LAG-3 (48). However, additional validation of these immune checkpoints by RT-qPCR, more detailed characterization of their expression in different cell types present in the biopsies, or assessment of the functional impact of these expression changes in immune checkpoints were not reported. Together, these reports suggest that vaping can alter the expression of immune checkpoints.

*In vitro* analysis indicated that exposure of human neutrophil to e-cigarette aerosols impaired migration, phagocytosis, oxidative burst response, extracellular trap formation, and induced changes in PD-L1 expression. Treatment with nicotine-containing e-cigarette aerosols increased PD-L1 expression (**Figure 1**) compared with nicotine-free treatment containing only the base elements propylene glycol and vegetable glycerin (49). The effects on the expression of additional immune checkpoints and on biological functions in these cells remain to be analyzed.

Additionally, *in vitro* and human studies have shown that e-cigarette aerosols can induce changes of PD-L1 expression in monocytes (50, 51). The THP-1 monocyte cell line treated with aqueous extracts obtained by dissolving e-cigarette aerosols in culture medium showed higher PD-L1 expression (**Figure 1**) compared with controls. Interestingly, the presence of nicotine in the aqueous extracts did not promote PD-L1 expression (51). Exposure to e-cigarette aerosols also induced alterations in cytokine and chemokine expression in these cells (51). However, the resulting biological effects of these alterations were not addressed.

Conversely, e-cigarette use has been reported to affect the expression of monocytic PD-L1 in human subjects exposed to physical stress. Circulating classical (CD14^++^CD16^−^) and intermediate monocytes (CD14^++^CD16^+^) showed increased PD-L1 expression after physical stress in non-smoking participants. However, prior nicotine use through vaping prevented this stress-induced increase in PD-L1 expression (**Figure 1**) (50). Mechanistically, oxygen deficiency related to physical stress could be associated with increased levels of HIF-1α, a transcription factor that can bind the promoter region of the PD-L1 gene and promote its expression (50, 52). Additionally, exercise has been suggested to reduce systemic inflammation, increase the frequency of PD1+CD8+T cells, and elevate levels of soluble PD-L1. These immune checkpoints may contribute to the maintenance of physiological health (50, 53). However, exposure to nicotine-containing e-cigarette aerosols disrupts the immune responses normally promoted by exercise. Collectively, these studies suggest that e-cigarette use can alter immune responses to various stimuli, such as viral infection or physical stress; moreover, modulation of immune checkpoints by e-cigarette aerosol components may be involved in these alterations.

Interestingly, exposure to e-cigarette aerosols promotes tumor growth and aggressive metastasis in a tumor mouse model (54). In this model, mice were exposed to aerosolized e-cigarette liquids containing propylene glycol and vegetable glycerin, with and without nicotine, and subsequently inoculated with tumor cells. Reduced survival rates, increased tumor growth, and higher counts of metastatic lung nodules were observed in all exposure groups compared with the control group (air-exposed mice inoculated with tumor cells) (54). These observations were attributed to immunosuppression promoted by e-cigarette aerosols. Specifically, exposure to aerosolized e-cigarette liquids containing propylene glycol and vegetable glycerin with and without nicotine led to increased accumulation of PD1+CD8+CD3+ T cells with impaired cytotoxic and proliferative capacities in the lungs of the tumor mouse model (**Figure 1**) (54). Analysis of immune checkpoints CTLA-4, PD-1, TIM-3, and LAG-3 on splenic CD8+CD3+ T cells in these mice showed that aerosols of e-cigarette liquids containing propylene glycol and vegetable glycerin with nicotine promoted higher expression of TIM-3 and CTLA-4 (**Figure 1**). In contrast, aerosols of e-cigarette liquids containing propylene glycol and vegetable glycerin without nicotine promoted higher expression of PD-1 compared with the control group (**Figure 1**). LAG-3 expression was not altered by exposure to e-cigarette aerosols (54). Furthermore, exposure to aerosolized e-cigarette liquids containing propylene glycol and vegetable glycerin with and without nicotine increased the accumulation of splenic CD8+ CD3+ T cells co-expressing the four checkpoints (CTLA-4, PD-1, TIM-3, and LAG-3) (**Figure 1**) (54). These findings suggest that the immunosuppressive microenvironment and the local and systemic accumulation of exhausted T cells expressing increased levels of immune checkpoints induced by e-cigarette aerosols may promote tumor progression. Collectively, these reports indicate that e-cigarette aerosols can alter the expression of diverse immune checkpoints in neutrophils, monocytes, and T cells. Changes in immune checkpoint expression depended on the composition of e-cigarette liquid and were associated with the promotion of important biological effects, such as metastasis and tumor growth, in the context of a cancer model.

### 4.2 E-cigarette compound: nicotine

#### 4.2.1 Nicotine and PD-L1

Melanoma cells treated with nicotine showed increased proliferation, migration, and PD-L1 expression in a time- and dose-dependent manner (55). Mechanistically, it was suggested that nicotine binds to the α9-nAChR (nicotinic acetylcholine receptor), promoting its activation and expression, which results in increased levels of phosphorylated signal transducer and activator of transcription 3 (STAT3). This transcription factor can translocate to the nucleus, bind the promoter region of the PD-L1 gene, and induce its expression in melanoma cells (55). Furthermore, nicotine has been suggested to bind to α5-nAChR and promote PD-L1 expression through a similar mechanism in lung cancer (56, 57). In this case, STAT3 binds the protomer regions of PD-L1 and JAB1, stimulating their expression. Importantly, JAB1 prevents ubiquitination and degradation of PD-L1, thereby mediating stabilization (57). These observations were supported by *in vitro* studies and xenograft tumor mouse models (56, 57). The resulting biological effects included reduced cytotoxic activity of T cells and the generation of CD4+CD25+FOXP3+ regulatory T cells with reduced production of IFNγ and IL-2 (57). Moreover, tumor mouse model inoculated with tumor cells overexpressing α5-nAChR showed increased tumor growth compared with the control group; however, treatment with BMS-1, a small-molecule inhibitor of the PD-1/PD-L1 interaction, reduced tumor growth in this model (57). Taken together, these observations suggest that regulation of PD-L1 by nAChR receptors and nicotine facilitates tumor immune escape. Additionally, increased PD-L1 expression mediated by nicotine-α5−nAChR promoted increased proliferation, migration, invasion, and expression of EMT markers (Zeb1, *N*-cadherin, vimentin and Snail1) in non-small cell lung cancers (56). Consistently, an independent report indicated that chronic nicotine treatment increased the expression of PD-L1 and α1-nAchR in a non-small cell lung cancer cell line (58). Nicotine also promoted epidermal growth factor receptor (EGFR) expression and activation of mTOR and Akt pathways. Importantly, nicotine induced relative resistance to treatment with the EGFR tyrosine kinase inhibitor gefitinib (58). It was suggested that PD-L1 could be a therapeutic target in cases of poor response to EGFR tyrosine kinase inhibitors under certain conditions (58). Collectively, these reports suggest that augmented PD-L1 expression in cancer cells not only enables immune evasion but also contributes to tumor growth, migration, and invasion (55, 56), with important implications for therapeutic strategies. Thus, nicotine may alter antitumor immunity, metastasis, and therapy response through modulation of this immune checkpoint in the cancer context.

In contrast, nicotine treatment was reported to reduce expression of PD-L1 and PD-L2 in SK-BR-3 breast cancer cells by reducing the levels of phosphorylated Akt (59). This effect was also associated with reduced expression of Wnt5a. Interestingly, these effects were not observed in other breast cancer cell lines (HCC1937 and MDA-MB-231), which are classified as triple-negative breast cancer (TNBC) cells [estrogen receptor (ER), progesterone receptor (PR) and human epidermal growth factor receptor 2 (HER2) negative] and are associated with poor prognosis (59). Conversely, SK-BR-3 is a HER2-positive cell line associated with intermediate prognosis. It was suggested that nicotine treatment could be beneficial in therapeutic strategies for this particular subtype of breast cancer (59). However, additional functional analyses and animal model studies addressing the biological effects resulting from altered expression of PD-L1 and PD-L2 are needed to strengthen these observations.

#### 4.2.2 Nicotine and PD-1

Culture of CD8+ T cells from patients with rheumatoid arthritis, stimulated with anti-CD3 antibodies and treated with nicotine, showed reduced PD-1 expression compared with cells not exposed to nicotine. It was suggested that this could alter the regulation of immunity (60). Consistently, an arthritic mouse model treated with nicotine showed a reduced percentage of PD-1+CD8+ T cells in bone marrow. Furthermore, nicotine promoted the generation of CD8+ T cells with a non-exhausted PD-1^-^IL-7R^+^ phenotype (61). These findings suggest that nicotine can contribute to loss of tolerance associated with the development of rheumatoid arthritis (61). In contrast, CD8+ T cells from healthy volunteers treated with nicotine showed reduced cytotoxic capacity against tumor cells in coculture, accompanied by increased PD-1 expression (62). Similarly, nicotine has been shown to induce exhausted T cells with reduced capacity to inhibit tumor viability and growth (62). Taken together, these reports indicate that nicotine regulates PD-1 expression in T cells, with biological implications for the development of different illnesses. Moreover, this differential regulation may be influenced by the specific microenvironment or disease context. It is possible that T cells behavior may be modulated by the presence or absence of a particular inflammatory environment. For example, differential response to nicotine might arise from variations in receptors expression; in this sense, it was reported the increased expression of the nicotine receptor α7-nAChR in different cells of animal models with rheumatoid arthritis (63–65). Furthermore, PD-1 expression can be influenced by changes in the expression or activation of different transcription factors such as STAT3 and T-bet (66) and aberrant signalling of these transcription factors have been reported in rheumatoid arthritis (67, 68). However, the impact of these alterations on nicotine-mediated PD-1 regulation remains to be determined. More studies analyzing the molecular mechanisms involved could help explain these apparent discrepancies.

#### 4.2.3 Nicotine and CTLA-4

Mouse regulatory T cells (CD4+CD25+ cells) pre-treated with nicotine exhibited increased suppressive activity compared with regulatory T cells without nicotine treatment, reducing proliferation and IL-2 production by stimulated T cells *in vitro*. Moreover, this enhanced suppressive capacity of regulatory T cells was associated with increased expression of CTLA-4 and Foxp3. The molecular mechanism involved stimulation of the α7-nAChR receptor (69), which can bind nicotine (70). It was suggested that the increased expression of CTLA-4 by regulatory T cells was crucial for inducing the observed immune suppression (69). An independent study reported that cultures of stimulated splenic mouse mononuclear cells treated with nicotine showed reduced generation of CTLA-4+ CD4+ T cells and decreased production of INFγ and IL-2 compared with control cultures without nicotine treatment *in vitro* (71). Together, these reports suggest that nicotine can modulate CTLA-4 expression in specific subtypes of T cells and promote immunosuppressive effects. The biological implications of these phenomena in more complex systems, such as animal models or disease conditions, remain to be determined.

Interestingly, the importance of CTLA-4 in the context of cancer and nicotine exposure can be observed indirectly in a tumor model (54). Pre-conditioned tumor cells generated by previous exposure to propylene glycol/vegetable glycerin and nicotine were inoculated subcutaneously into mice, producing solid tumor masses and metastases (54). Treatment with an anti-CTLA-4 antibody reduced tumor growth and metastasis in this model. In this sense, it was suggested that pre-conditioning of tumor cells with the main components of e-cigarette accelerates tumor progression and that e-cigarette aerosols can promote CTLA-4 expression in T cells in the cancer context (54). Thus, further studies are needed to determine the molecular mechanisms involved in the regulation of immune checkpoints, such as CTLA-4, by e-cigarette aerosols in the cancer context.

#### 4.2.4 Nicotine and other immune checkpoints

Nicotine can promote CD47 expression in human non-small cell lung cancer cell lines (72) through a mechanism similar to that used to modulate other immune checkpoints. Nicotine activates and induces expression of the α5-nAChR receptor in these tumor cells, resulting in increased levels of phosphorylated STAT3. This transcription factor binds the promoter region of CD47, inducing its expression (72). Importantly, activation of this pathway promoted proliferation, migration, and invasion of tumor cells *in vitro*, including stem cell-like characteristics, such as increased levels of CD44, SOX2, and NANOG and enhanced sphere formation efficiency (72). Moreover, a mouse cancer model indicated activation of this axis by nicotine (72). *In vitro* studies suggested that activation of the nAChR/STAT3/CD47 axis in tumor cells reduced phagocytosis by monocytes and decreased TNFα levels. Consistently, mouse models inoculated with tumor cells overexpressing α5-nAChR showed increased tumor growth and elevated levels of SOX2, CD47, and phosphorylated STAT3. However, treatment with a CD47 inhibitor reverted these effects, highlighting the importance of immune checkpoint regulation by nicotine in the cancer context and its potential therapeutic relevance (72).

Additionally, nicotine has been reported to reduce CD24 expression in a lung cancer cell line, involving increased Ras levels (73). However, the biological effects and underlying molecular mechanisms were not determined.

## 5 Discussion

Given that the global market for e-cigarettes has expanded considerably, which is related to their increased popularity (16), it is essential to analyze the biological effects of vaping. A review of the role of aerosols generated by e-cigarettes or their main components on the regulation and functions of immune checkpoints has not been previously conducted. This is relevant due to the capacity of immune checkpoints to modulate immune responses under conditions of health and in the context of different diseases (74). In this sense, e-cigarette aerosols can modulate the expression of immune checkpoints in nasal epithelial cells, monocyte subtypes, and neutrophils from healthy individuals (47–50). Some of these expression changes were observed in association with alterations in immune responses to physical stress and viral infections in healthy e-cigarette users (47, 48, 50). However, the actual contribution of these expression changes to biological effects was not addressed. Future studies should determine whether e-cigarette aerosols can induce susceptibility to infections or increase the risk of developing certain diseases through mechanisms involving modulation of immune checkpoints.

Suppressive capacity of immune checkpoints has been analyzed principally in cancer and as targets in immunotherapies (74). In view of this, e-cigarette aerosol promoted tumor development in a mouse model (54). The immunosuppressive response was related to cancer promotion by e-cigarette aerosols, with increased expression of PD-1, CTLA-4, and TIM-3, as well as increased accumulation of T cells expressing PD-1, CTLA-4, LAG-3, and TIM-3 (54). This suggests that e-cigarettes can exert suppressive effects through the regulation of immune checkpoints, resulting in cancer promotion in this model. Additional studies could determine the molecular mechanisms involved in this regulation or implications in immunotherapies.

Moreover, immune checkpoints can regulate other biological process in tumors, such as self-renewal, epithelial-mesenchymal transition, metastasis, drug resistance, anti-apoptosis, angiogenesis, or metabolism (75). In this context, it was not determined whether the increase in PD-1, CTLA-4, and LAG-3 expression mediated by e-cigarette aerosols promoted any of the aforementioned biological processes. Although long-term health effects of vaping have not been well determined, it has been suggested that e-cigarettes increase the risk of certain types of cancer (76). Thus, additional studies could determine the contribution of immune checkpoints to the development of different types of cancer during e-cigarette exposure.

Interestingly, e-cigarette use has been associated with other illnesses, such as periodontal diseases (77) and chronic obstructive pulmonary disease (78), and these diseases have shown alterations in the function and expression of diverse immune checkpoints (79, 80). Whether vaping can regulate the function of immune checkpoints and exert biological effects in the context of different diseases associated with its use remains to be determined.

The composition of aerosols generated by heating e-cigarette liquid is related to the constituents of the liquid (16). In this sense, studies analyzing the effect of e-cigarette aerosols on immune checkpoints showed that the composition of e-liquids generated differential effects on immune checkpoint expression (54). Interestingly, aerosols generated from e-liquids containing only propylene glycol and/or vegetable glycerin promoted tumor development and accumulation of T cells expressing PD-1, CTLA-4, LAG-3, and TIM-3, which was associated with immunosuppression (54).

Even though propylene glycol and vegetable glycerin are included in the Generally Recognized as Safe list by the United States Food and Drug Administration (5), when aerosolized they may have harmful biological effects, such as cytotoxicity and genotoxicity in oral keratinocytes (81) or immunosuppression and cancer promotion meditated by modulation of immune checkpoints (54). Additionally, operational parameters such as vaporization temperature, aspiration flow rate, or the utilization of coils and wicks affect the composition of aerosols generated by heating e-cigarette liquid (16). Studies analyzing the effect of e-cigarettes on immune checkpoints while considering these parameters are absent. This could generate a more detailed picture of the biological effects resulting from the use of electronic cigarettes and resolve possible discrepancies in the reports.

The main components of e-liquids include propylene glycol, vegetable glycerin, nicotine, and flavoring agents (28). A search and analysis of the available information to determine whether these compounds modulate inhibitory immune checkpoints revealed that most published studies focus on analyzing nicotine-mediated regulation of diverse immune checkpoints. On the other hand, to the best of our knowledge, reports analyzing the roles of the humectants present in most e-liquids, propylene glycol and glycerin, on inhibitory immune checkpoints are absent.

Nicotine can modulate the expression of PD-L1, PD-L2, PD-1, CTLA-4, CD47, and CD24 in diseases such as melanoma, lung cancer, breast cancer, and rheumatoid arthritis. The molecular mechanisms regulating the expression of some of these immune checkpoints involve different subtypes of nAChR receptors, which activate the STAT3 pathway (55–60, 69, 71–73). Whether these molecular mechanisms are involved in the regulation of immune checkpoint expression by e-cigarette aerosols has not been determined.

Interestingly, nicotine has been demonstrated to regulate processes such as immune evasion, proliferation, metastases, and stem cell-like characteristics via immune checkpoints in association with nAChR receptors in the cancer context (55–57, 72). This suggests that nicotine has a major impact on tumor development mediated by immune checkpoints, at least in the described models. This was also supported by findings showing that inhibitors of immune checkpoints reverted the pro-tumoral effects (54, 57, 72), suggesting possible therapeutic applications. The effects of e-cigarette aerosols and core e-liquid components on the regulation of inhibitory immune checkpoint expression and functions derive principally from *in vitro* and murine models. While these models are essential for dissecting molecular mechanisms, the direct translation of these findings to e-cigarette users remains limited. The analysis of immune checkpoints in *ex vivo* models with cells from e-cigarette users could clarify the actual impact of these findings on human health. Furthermore, the impact of short- and long-term e-cigarette aerosol inhalation should be considered in these experimental models as biological effects differ significantly between acute exposure and chronic inhalation (82). Additionally, evaluating immune checkpoint profiles in human populations exposed to e-cigarettes may identify their potential utility as biomarkers.

The availability of hundreds or thousands of e-liquid flavors, oscillating from basic formulas such as menthol or tobacco to fruit and candy varieties, is related to the popularity of e-cigarettes (28). It has been suggested that flavors could motivate adult cigarette users to switch to a less harmful product to diminish health risk; on the other hand, they may encourage young people and non-users to vape (83). Analyses of dataset from the European Common Entry Gate system found 213 different flavoring agents reported to be added to diverse e-cigarette liquids. The most recurrently incorporated flavoring agents were vanillin, ethyl maltol, and ethyl butyrate (84). To the best of our knowledge, reports analyzing the modulation of expression or functional regulation of inhibitory immune checkpoints by these particular flavoring agents are lacking.

Additionally, it has been reported that a variety of flavoring agents can be harmful (83). A literature analysis identified that 65 flavoring agents added to diverse e-liquids showed the capacity to induce toxic effects on the respiratory tract, cardiovascular and circulatory systems, skeletal system, and skin (85). Cinnamaldehyde was most repeatedly described flavoring agent to be cytotoxic (85). Evidence supports the toxicological impact of cinnamaldehyde-containing e-liquids following inhalation (86). Interestingly, this flavoring ingredient can regulate the expression of the inhibitory immune checkpoint PD-L1 (87, 88). Dendritic cells derived from human monocytes, the THP-1 monocyte cell line, and the MUTZ-3 human cytokine–dependent myeloid cell line treated with cinnamaldehyde increased expression of PD-L1 (87, 88). Also, increased expression of other markers and cytokines was observed; these findings were related to the capacity of cinnamaldehyde to induce skin sensitization (87), as it is considered a moderate sensitizer (89). Notably, these findings may not accurately reflect pulmonary exposure. Consequently, the impact of cinnamaldehyde on PD-L1 expression in lungs following inhalation remains to be established. Biological impact using more complex systems, molecular mechanisms involved in regulation of PD-L1, and modulation of others immune checkpoints by this flavoring agent remain to be determined. Diacetyl, a flavoring agent linked to specific pulmonary problems, can be present in e-cigarettes (90, 91). To the best of our knowledge, reports analyzing the expression or functional regulation of inhibitory immune checkpoints by diacetyl are absent. Diacetyl inhalation has been strongly associated with the development of bronchiolitis obliterans, a destructive fibrotic lung disease (92) with airway inflammation and peribronchial fibrosis (93). Interestingly, bronchiolitis obliterans also can be caused by lung transplantation and severe respiratory tract infections (92). Inhibitory immune checkpoints have been analyzed in bronchiolitis obliterans syndrome (94), a clinical manifestation of bronchiolitis obliterans in patients who have undergone lung transplantation (95). PD-1, PD-L1, and CTLA-4 are expressed on T cells within the lung tissues of patients with bronchiolitis obliterans syndrome (94). However, expression patterns or roles of inhibitory immune checkpoints in bronchiolitis obliterans caused by diacetyl have not been determined. Additionally, more studies are necessary to determine the contribution of frequent and uncommon flavoring ingredients in e-liquids to the regulation of immune checkpoints, since these can modulate immune response in different disease contexts.

In conclusion, e-cigarette aerosols, as well as e-liquid compounds such as nicotine and flavoring agents, have the capacity to modulate the expression and function of diverse inhibitory immune checkpoints in models of different types of cancer, rheumatoid arthritis, and physical stress. More studies are necessary to determine the biological effects generated by alterations in the immune checkpoint expression mediated by e-cigarette aerosols and their real contribution to disease development.
